# High-level extracellular protein production in *Bacillus subtilis* using an optimized dual-promoter expression system

**DOI:** 10.1186/s12934-017-0649-1

**Published:** 2017-02-20

**Authors:** Kang Zhang, Lingqia Su, Xuguo Duan, Lina Liu, Jing Wu

**Affiliations:** 1 State Key Laboratory of Food Science and Technology, Jiangnan University, 1800 Lihu Avenue, Wuxi, 214122 China; 2 School of Biotechnology and Key Laboratory of Industrial Biotechnology Ministry of Education, Jiangnan University, 1800 Lihu Avenue, Wuxi, 214122 China

**Keywords:** *Bacillus subtilis*, High-level expression, Promoter optimization, General applicability, Scale-up production

## Abstract

**Background:**

We recently constructed a *Bacillus subtilis* strain (CCTCC M 2016536) from which we had deleted the *srfC*, *spoIIAC*, *nprE*, *aprE* and *amyE* genes. This strain is capable of robust recombinant protein production and amenable to high-cell-density fermentation. Because the promoter is among the factors that influence the production of target proteins, optimization of the initial promoter, P_amyQ_ from *Bacillus amyloliquefaciens*, should improve protein expression using this strain. This study was undertaken to develop a new, high-level expression system in *B. subtilis* CCTCC M 2016536.

**Results:**

Using the enzyme β-cyclodextrin glycosyltransferase (β-CGTase) as a reporter protein and *B. subtilis* CCTCC M 2016536 as the host, nine plasmids equipped with single promoters were screened using shake-flask cultivation. The plasmid containing the P_amyQ′_ promoter produced the greatest extracellular β-CGTase activity; 24.1 U/mL. Subsequently, six plasmids equipped with dual promoters were constructed and evaluated using this same method. The plasmid containing the dual promoter P_HpaII_–P_amyQ′_ produced the highest extracellular β-CGTase activity (30.5 U/mL) and was relatively glucose repressed. The dual promoter P_HpaII_–P_amyQ′_ also mediated substantial extracellular pullulanase (90.7 U/mL) and α-CGTase expression (9.5 U/mL) during shake-flask cultivation, demonstrating the general applicability of this system. Finally, the production of β-CGTase using the dual-promoter P_HpaII_–P_amyQ′_ system was investigated in a 3-L fermenter. Extracellular expression of β-CGTase reached 571.2 U/mL (2.5 mg/mL), demonstrating the potential of this system for use in industrial applications.

**Conclusions:**

The dual-promoter P_HpaII_–P_amyQ′_ system was found to support superior expression of extracellular proteins in *B. subtilis* CCTCC M 2016536. This system appears generally applicable and is amenable to scale-up.

**Electronic supplementary material:**

The online version of this article (doi:10.1186/s12934-017-0649-1) contains supplementary material, which is available to authorized users.

## Background

*Bacillus subtilis*, a well-studied Gram-positive bacterium, has many outstanding features. It is non-pathogenic [1], has superior protein secretory capability, and has excellent biochemical and physiological characteristics. Downstream purification of secreted heterologous proteins is relatively easy because the proteins are harvested from the culture medium [2]. Therefore, systems that direct the extracellular expression of heterologous proteins have been used extensively for the efficient production of industrial enzymes, antibiotics, and medicinal proteins [3].

Over the years, efforts to improve and optimize the *B. subtilis* expression system have mainly involved strain modification and expression plasmid construction. Many strains deficient in exoenzyme or exoprotease production have been constructed to minimize the expression of unwanted exoenzymes and protein degradation [4]. For example, WB600, derived from *B. subtilis* 168, is a strain deficient in six proteases [4]. At the same time, expression plasmids have been modified to enhance protein expression in *B. subtilis*. Because the promoter was found to be among the elements that influence target gene transcription, several approaches have been used to identify novel promoters, including the screening of chromosomal DNA fragments [5], the modification of conserved promoter sequences [6], and the construction of two or more tandem promoters [7]. Excellent expression plasmids have been developed with efficient promoters like the *B. subtilis* T7 expression system promoter P_spac_ [8], the *B. subtilis* sucrose-inducible promoter P_sacB_ [9], the *B. megaterium* [10] and *B. subtilis* [11] xylose-inducible promoter P_xylA_, the *Bacillus amyloliquefaciens* α-amylase promoter P_amyQ_ [12], the *Staphylococcus aureus* constitutive strong promoter P_HpaII_ [13] and the *B. subtilis* autoregulatory promoter P_srfA_ [14]. An excellent example of these efforts is the use of the α-amylase promoter P_amyQ_ from *B. amyloliquefaciens* to express cycloisomaltooligosaccharide glucanotransferase in a protease-deficient *B. subtilis* strain. Using this system, expression can satisfy the demands of industrial applications [12]. Similarly, the dual-promoter P_gsiB_–P_HpaII_ system has been used to overproduce aminopeptidase in 5-L fermenter, resulting in the production of 205 U/mL (1.7 g/L) [7].

The widely used industrial enzymes α-cyclodextrin glycosyltransferase (α-CGTase), β-cyclodextrin glycosyltransferase (β-CGTase), and pullulanase are obtained through extracellular expression [15, 16]. α-CGTase and β-CGTase are primarily used in the enzymatic production of α- and β-cyclodextrins, which are cyclic oligomers of glucose residues linked by α-1,4-glycosidic bonds. These compounds are widely used in the food, cosmetics, pharmaceutical, and chemical industries [17]. CGTases have been expressed in *Escherichia coli* [18], *B. subtilis* [19], *B. circulans* ATCC 21783 [20], alkalophilic *Bacillus* sp. TS1-1 [21] and *B. macerans* [22]. Unfortunately, these systems suffer from issues related to food safety or low expression levels. Pullulanase is a well-known debranching enzyme that cleaves the α-1,6 glycosidic linkages in pullulan, amylopectin, and the α- and β-limit dextrins of amylopectin. This enzyme can be used alone or in conjunction with other amylolytic enzymes (α-amylase, β-amylase, glucoamylase, or CGTase) to break down starch; the products include small reducing sugars, cyclodextrins, and amylose [23]. Pullulanase production has been well-studied in recent years because of its extensive application in the food and chemical fuel industries [24]. Pullulanase has been expressed in *E. coli* [23], *B. subtilis* [25], *B. flavothermus* [26], *B. licheniformis* [27], *Brevibacillus choshinensis* [24], *Pichia pastoris* [28] and *Saccharomyces cerevisiae* [29]. Expression levels are high in *E. coli,* but the use of *E. coli* is restricted in industrial food applications because of its potential pathogenicity. Unfortunately, expression levels in the other hosts are poorer, and cannot satisfy industrial needs. For the reasons stated above, increasing the expression of these three enzymes in *B. subtilis* has high industrial value.

In previous work, we constructed *B. subtilis* strain CCTCC M 2016536 from an undomesticated *B. subtilis* by deleting the *srfC*, *spoIIAC*, *nprE*, *aprE* and *amyE* genes. Protein production using this strain is superior to that of common model laboratory strains. A construct consisting of β-CGTase from *Bacillus circulans* 251 fused to the signal peptide amyQ was expressed in *B. subtilis* CCTCC M 2016536 using the amylase promoter P_amyQ_ from *B. amyloliquefaciens*, and good levels of expression were demonstrated in a 3-L fermenter. In this work, we constructed nine single-promoter plasmids and six dual-promoter plasmids using a combinatory approach. Then, with β-CGTase, pullulanase, and α-CGTase as reporter proteins and *B. subtilis* CCTCC M 2016536 as the expression host, we evaluated the levels of extracellular protein expression using these promoters in shake-flask experiments. The dual promoter P_HpaII_–P_amyQ′_ mediated the highest extracellular expression of β-CGTase, as well as high-level extracellular expression of pullulanase and α-CGTase. Expression of β-CGTase using the dual-promoter P_HpaII_–P_amyQ′_ system was subsequently scaled up using a 3-L fermenter. The results of these experiments demonstrate that this new expression system has high potential for use in industrial applications.

## Results and discussion

### Optimization of promoters for β-CGTase expression

The promoter is one of the factor that influence the transcription of target protein and its optimization was seen as an efficient method to improve expression of heterologous proteins. The plasmid pHYCGT1, which contains the β-CGTase gene from *Bacillus circulans* 251, the amylase promoter P_amyQ_ and the signal peptide amyQ from *B. amyloliquefaciens*, was used as the initial β-CGTase expression plasmid in *B. subtilis* CCTCC M 2016536. Because of their recognized ability to drive target protein expression in *B. subtilis*, the five widely used promoters P_srf_ [14], P_xyl′_ [11], P_gsiB_ [30], P_xyl_ [10] and P_HpaII_ [13] (Table 1) were chosen to replace the P_amyQ_ promoter of plasmid pHYCGT1. These replacements yielded plasmids pHYCGT2, pHYCGT3, pHYCGT4, pHYCGT5 and pHYCGT6, respectively (Table 2).

**Table 1:** Properties of promoters used for β-CGTase expression optimization

Promoter	Origin	Properties	Expression reporter proteins
P_srf_	*B. subtilis*	Auto-inducible system regulated by ComA–ComP phosphorylation system [14]	Green florescent protein, aminopeptidase
P_xyl′_	*B. subtilis*	Xylose-based expression system and catabolite repressed by catabolite-responsive element [11]	β-Galactosidase, glycerol-3-phosphate cytidylyltransferase
P_gsiB_	*B. subtilis*	Subject to σ^B^ regulation and is induced by ethanol, heat and acid shock [30]	β-Galactosidase [50]
P_xyl_	*B. megaterium*	Xylose-based expression system and glucose repression [10]	β-Galactosidase and other heterologous proteins
P_HpaII_	*Staphylococcus aureus*	Strong constitutive promoter that stimulates counterclockwise RNA synthesis [13]	β-Galactosidase, chloramphenicol acetyltransferase and other heterologous proteins
P_amyQ′_	*B. subtilis*	Regulated by the DegS–DegU two-component system [32]	β-Galactosidase
P_aprE_	*B. subtilis*	Promoter of alkaline protease	None
P_nprE_	*B. subtilis*	Promoter of neutral protease	None

**Table 2:** Plasmids

Plasmid	Description	Reference
pNCMO2/*pul*A-d2	*Brevibacillus choshinensis*–*E. coli* shuttle vector, Amp^r^ (*E. coli*), Ner^r^ (*Brevibacillus choshinensis*), pullulanase gene	[22]
pHY300PLK	*B. subtilis*–*E. coli* shuttle expression vector, Amp^r^ (*E. coli*), Tet^r^ (*B. subtilis* and *E. coli*)	Takara
pHYCGT1	*B. subtilis*–*E. coli* shuttle expression vector, Amp^r^ (*E. coli*), Tet^r^ (*B. subtilis* and *E. coli*), amylase promoter P_amyQ_ and signal peptide amyQ from *Bacillus amyloliquefaciens*, β-CGTase gene.	[28]
pET-20b(+)/*cgt*	*E. coli* gene expression vector, Amp^r^, α-CGTase gene	[29]

pHYCGT1 derivative	Promoter	Signal peptide	Reporter gene	Reference
pHYCGT2	P_srf_	amyQ	β-CGTase gene	This work
pHYCGT3	P_xyl′_	amyQ	β-CGTase gene	This work
pHYCGT4	P_gsiB_	amyQ	β-CGTase gene	This work
pHYCGT5	P_xyl_	amyQ	β-CGTase gene	This work
pHYCGT6	P_HpaII_	amyQ	β-CGTase gene	This work
pHYCGT7	P_amyQ′_	amyQ′	β-CGTase gene	This work
pHYCGT8	P_aprE_	aprE	β-CGTase gene	This work
pHYCGT9	P_nprE_	nprE	β-CGTase gene	This work
pHYCGTd1	P_srf_ and P_amyQ′_	amyQ′	β-CGTase gene	This work
pHYCGTd2	P_xyl′_ and P_amyQ′_	amyQ′	β-CGTase gene	This work
pHYCGTd3	P_gsiB_ and P_amyQ′_	amyQ′	β-CGTase gene	This work
pHYCGTd4	P_HpaII_ and P_amyQ′_	amyQ′	β-CGTase gene	This work
pHYCGTd5	P_amyQ′_ and P_amyQ′_	amyQ′	β-CGTase gene	This work
pHYCGTd6	P_nprE_ and P_amyQ′_	amyQ′	β-CGTase gene	This work
pHYPUL1	P_amyQ_	amyQ	Pullulanase gene	This work
pHYPUL7	P_amyQ′_	amyQ′	Pullulanase gene	This work
pHYPULd4	P_HpaII_ and P_amyQ′_	amyQ′	Pullulanase gene	This work
pHYαCGT1	P_amyQ_	amyQ	α-CGTase gene	This work
pHYαCGT7	P_amyQ′_	amyQ′	α-CGTase gene	This work
pHYαCGTd4	P_HpaII_ and P_amyQ′_	amyQ′	α-CGTase gene	This work

Considering that alpha amylase, alkaline protease AprE, and neutral protease NprE are among the most highly expressed extracellular *B. subtilis* proteins, the promoter regions from these three genes were also chosen for study. Because expression systems that use the promoter and signal peptide from the same gene show high-level extracellular production of the target protein [12, 31], the three promoters were evaluated with their own signal peptides. The promoter P_amyQ_ and signal peptide amyQ of plasmid pHYCGT1 were replaced with promoters P_amyQ′_, P_aprE_ and P_nprE_ (Table 1) and signal peptides amyQ′, aprE and nprE from *B. subtilis* CCTCC M 2016536, respectively, yielding plasmids pHYCGT7, pHYCGT8 and pHYCGT9 (Table 2).

**Fig. 1:**
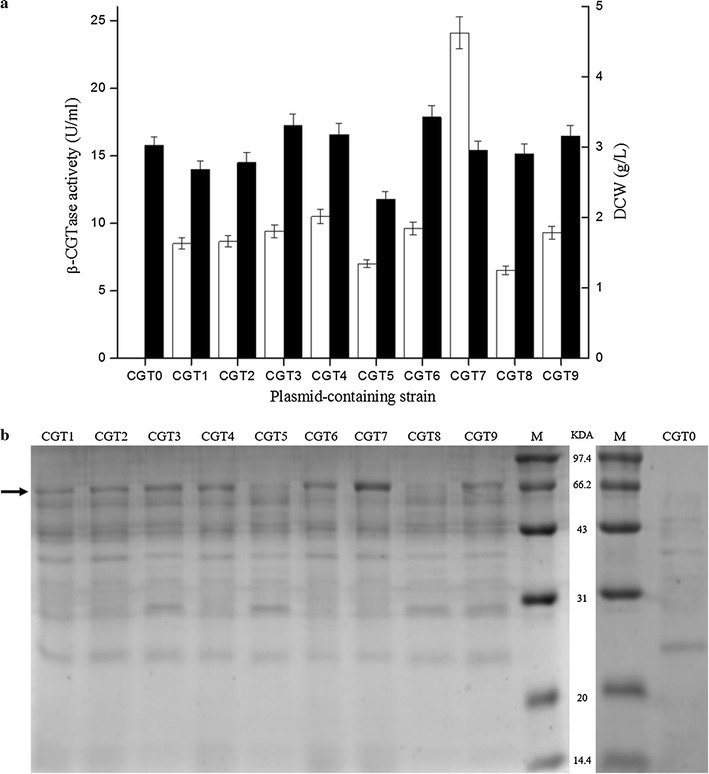
Extracellular β-CGTase expression driven by single-promoter systems in *B. subtilis* strains. Extracellular β-CGTase activity (*white*), dry cell weight (*black*) (**a**). SDS-PAGE analysis of extracellular β-CGTase expression by these plasmid-containing strains (**b**) (*P* < 0.05)

The nine plasmids described above were used to transform *B. subtilis* CCTCC M 2016536 in which the genes encoding alpha amylase, protease AprE and NprE are disrupted. These transformations created the nine corresponding plasmid-containing strains CGT1 through CGT9 (Additional file 1: Table S1). The relative strengths of these promoters were determined by measuring the extracellular β-CGTase activities of the nine plasmid-containing strains using shake-flask cultivation. Eight of the nine promoters, including promoter P_xyl′_, which does not contain the xylose repressor encoded by *xylR*, are constitutive promoters. P_xyl_, the only inducible promoter among the nine, was best induced with 5 g/L xylose. As shown in Fig. 1a, the extracellular β-CGTase activity of strains CGT1 through CGT9 were 8.5, 8.7, 9.4, 10.5, 7.0, 9.6, 24.1, 6.5 and 9.3 U/mL, respectively. The plasmid-containing strain CGT7, which harbors the plasmid containing promoter P_amyQ′_ and signal sequence amyQ′, showed the highest β-CGTase activity; almost 2.3- to 3.7-fold that of the other plasmid-containing strains. Moreover, the plasmid-containing strain CGT0, which carries the empty expression vector pHY300PLK, shows no β-CGTase activity after the same cultivation. SDS-PAGE analysis of supernatant proteins was carried out to verify these results. As shown in Fig. 1b, the thicknesses of the appropriate bands were in good agreement with the β-CGTase activity values. The dry cell weight of plasmid-containing strain CGT5 showed the lowest expression level (2.26 g/L) and the plasmid-containing strain CGT6 showed the highest expression level (3.43 g/L) (Fig. 1a).

### Optimization of dual promoters and promoter characteristics

Having compared nine single-promoter plasmids and identified promoters P_srf_, P_xyl′_, P_gsiB_, P_HpaII_, P_amyQ′_, and P_nprE_ as superior among them, with P_amyQ′_ being the strongest, dual-promoter plasmids were constructed to further increase β-CGTase expression. To create the dual-promoter constructs, promoters P_srf_, P_xyl′_, P_gsiB_, P_HpaII_, P_amyQ′_, and P_nprE_ were inserted into pHYCGT7 upstream of promoter P_amyQ′_, yielding plasmids pHYCGTd1, pHYCGTd2, pHYCGTd3, pHYCGTd4, pHYCGTd5 and pHYCGTd6, respectively (Table 2). The six dual-promoter plasmids were used to transform *B. subtilis* CCTCC M 2016536, yielding the six corresponding plasmid-containing strains CGTd1 through CGTd6 (Additional file 1: Table S1).

**Fig. 2:**
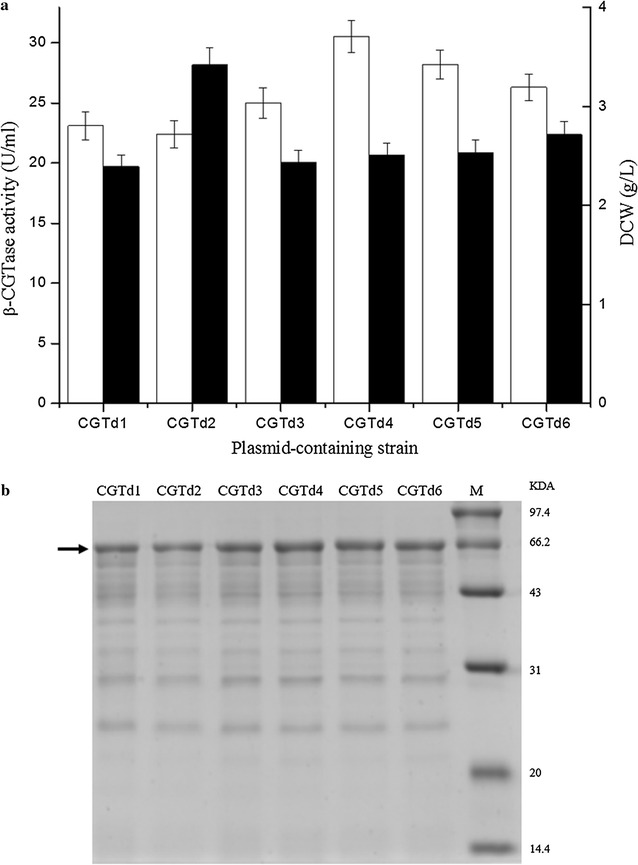
Extracellular β-CGTase expression driven by different dual-promoter systems in *B. subtilis* strains. Extracellular β-CGTase activity (*white*), dry cell weight (*black*) (**a**). SDS-PAGE analysis of extracellular β-CGTase expression by these plasmid-containing strains (**b**) (*P* < 0.05)

The expression strengths of the six dual promoters were measured using shake-flask experiments similar to those used to assess the single promoters. As shown in Fig. 2a, the extracellular β-CGTase activities of plasmid-containing strains CGTd1 through CGTd6 were 23.1, 22.4, 25.0, 30.5, 28.2 and 26.3 U/mL, respectively. The plasmid-containing strain CGTd4, which harbored the plasmid pHYCGTd4 with the dual promoter P_HpaII_–P_amyQ′_, showed the highest β-CGTase activity. This activity was almost 1.3-fold the activity produced by plasmid-containing strain CGT7, which expressed β-CGTase using the P_amyQ′_ promoter. These results were verified using SDS-PAGE analysis (Fig. 2b). The dry cell weight of plasmid-containing strain CGTd2 (3.42 g/L) was much higher than that of the other five plasmid-containing strains (Fig. 2a). In previous work, dual-promoter systems were constructed by inserting different single promoters downstream of a superior promoter; the expression strengths of these dual promoters differed greatly [7]. In this study, the six superior single promoters were inserted upstream of the strongest promoter, P_amyQ′_. The differences in expression strength shown by the dual-promoter systems were relatively weaker than the differences shown by the nine single-promoter systems. An explanation for this result might be that the transcriptional strength of a dual promoter depends largely on the strength of the promoter adjacent to the heterologous gene, and the expression strength of the downstream promoter P_amyQ′_ was much higher than that of the other five promoters P_srf_, P_xyl′_, P_gsiB_, P_HpaII_, and P_nprE_.

**Fig. 3:**
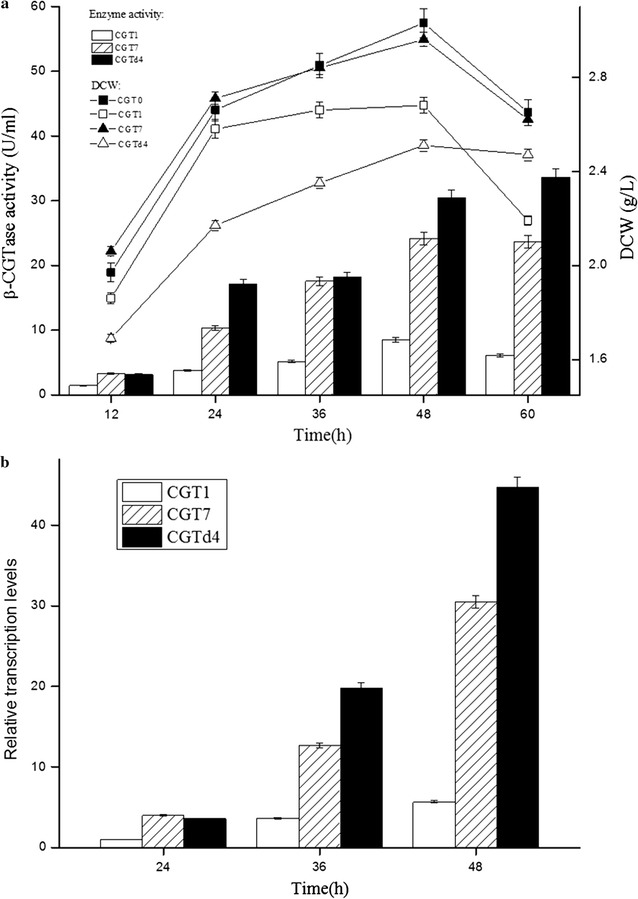
Extracellular β-CGTase expression and gene transcription level of plasmid-containing strains CGT1, CGT7 and CGTd4. Extracellular β-CGTase activity profile (*column*) and growth curve (*line chart*) of plasmid-containing strains CGT1, CGT7 and CGTd4 (**a**). Results of quantitative real-time PCR in plasmid-containing strains CGT1 (*white*), CGT7 (*sparse*) and CGTd4 (*black*) (**b**) (*P* < 0.05)

To investigate the β-CGTase expression characteristics mediated by promoters P_amyQ_, P_amyQ′_ and P_HpaII_–P_amyQ′_, we detected the β-CGTase production profiles and growth curves of plasmid-containing strains CGT0, CGT1, CGT7 and CGTd4. Plasmid-containing strain CGT0, which carried the empty expression vector pHY300PLK, showed no β-CGTase activity. As shown in Fig. 3a, the β-CGTase activities of plasmid-containing strains CGT1 and CGT7 peaked at 48 h, while the β-CGTase activity of plasmid-containing strain CGTd4 increased 10.3% from 48 to 60 h. The dry cell weights of plasmid-containing strains CGT0, CGT1, CGT7 and CGTd4 peaked at 48 h, while the dry cell weights of plasmid-containing strains CGT0 (12.5%), CGT1 (18.3%) and CGT7 (11.5%) decreased substantially from 48 to 60 h, compared with that of plasmid-containing strain CGTd4 (1.6%).

As for the specific enzyme activity of β-CGTase in *B. subtilis* WB600 was reported to be 231.6 U/mg [17], the β-CGTase expression level of CGT1, CGT7 and CGTd4 at 48 h was about 0.04, 0.10 and 0.13 mg/mL, respectively. To figure out if the β-CGTase expression was influenced by the transcriptional levels of promoters P_amyQ_, P_amyQ′_ and P_HpaII_–P_amyQ′_, the β-CGTase mRNA levels in plasmid-containing strains CGT1, CGT7 and CGTd4 were investigated using quantitative real-time PCR. As shown in Fig. 3b, the β-CGTase mRNA levels in plasmid-containing strains CGT7 and CGTd4 were similar at 24 h, but almost threefold greater than that of plasmid-containing strain CGT1. The β-CGTase mRNA levels in the plasmid-containing strains CGT7 (217.3%) and CGTd4 (455%) increased substantially from 24 to 36 h. The β-CGTase mRNA level of plasmid-containing strain CGTd4 was 1.6-fold greater than that of CGT7 and 5.4-fold greater than that of CGT1 at 36 h. The β-CGTase mRNA level in plasmid-containing strain CGTd4 was 1.5-fold greater than that of CGT7 and 7.9-fold greater than that of CGT1 at 48 h. These results demonstrate that the transcription strength of dual-promoter P_HpaII_–P_amyQ′_ was somewhat stronger than that of promoter P_amyQ_ and much stronger than that of promoter P_amyQ′_.

**Fig. 4:**
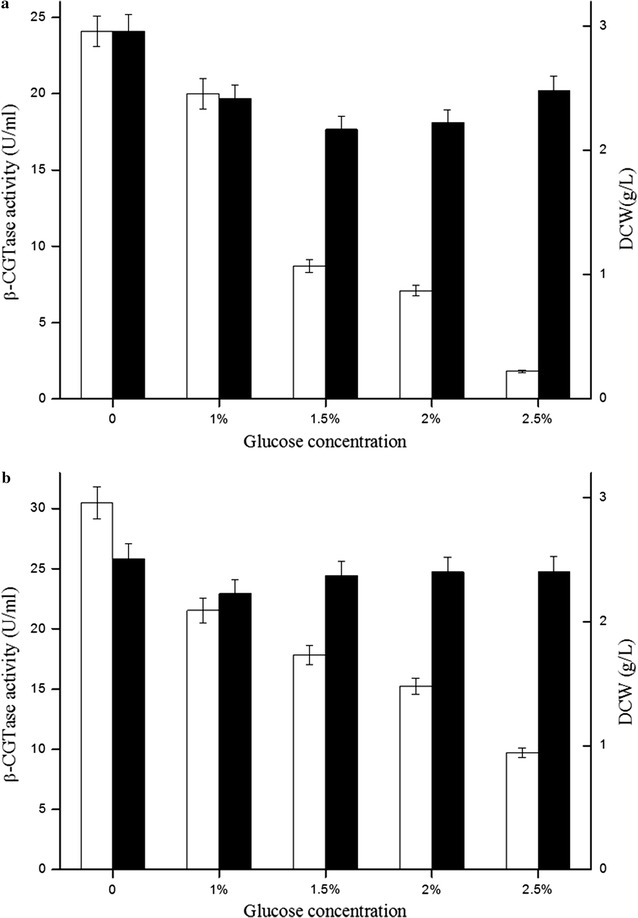
Extracellular β-CGTase expression by plasmid-containing strains CGT7 (**a**) and CGTd4 (**b**) with glucose addition. Extracellular β-CGTase activity (*white*), dry cell weight (*black*) (*P* < 0.05)

The promoter P_amyQ′_ contains a specific binding sequence for the DegS–DegU system, which is a two-component system that regulates the transcription of degradative enzyme genes in *Bacillus subtilis* [32]. To investigate whether single promoter P_amyQ′_ and dual promoter P_HpaII_–P_amyQ′_ were autoregulatory, the β-CGTase expression levels of plasmid-containing strains CGT7 (P_amyQ′_ promoter) and CGTd4 (dual promoter P_HpaII_–P_amyQ′_) were investigated with added glucose. As shown in Fig. 4, the extracellular β-CGTase activities of plasmid-containing strains CGT7 and CGTd4 gradually decreased with increasing concentrations of added glucose. Moreover, the dry cell weights of both plasmid-containing strains grown with added glucose were slightly reduced at 48 h. The dry cell weight of the plasmid-containing strain CGT7 was the lowest (2.17 g/L) at 48 h when grown in the presence of 1.5% added glucose, decreasing about 20% compared with the dry cell weight seen without addition of glucose (Additional file 1: Figure S1). The dry cell weights in the presence of 2 and 2.5% added glucose were 2.23 and 2.48 g/L, respectively. This is higher than the dry cell weight seen with 1.5% added glucose (2.17 g/L), which may result from their relatively lower β-CGTase expression (Additional file 1: Figure S1). These results demonstrate that the single promoter P_amyQ′_ and the dual promoter P_HpaII_–P_amyQ′_ were somewhat glucose repressed, and the degree of repression was enhanced with increasing glucose concentration. This indicates that the glucose concentration should be controlled during fermentation process.

In this study, the extracellular β-CGTase activity of plasmid-containing strain CGTd4 was not the simple addition of the results seen with plasmid-containing strains CGT6 and CGT7, which harboring single promoters P_HpaII_ and P_amyQ′_, respectively. Moreover, P_HpaII_ is a strong promoter and its constitutive transcription during the growth stage applied metabolic pressure to the growing cells. This pressure had a negative effect on total β-CGTase expression. The P_amyQ′_ promoter and the dual P_HpaII_–P_amyQ′_ promoter were somewhat glucose repressed and their transcription strengths were relatively weaker at 24 h compared with 36 and 48 h, as described above. Because there are more σ^A^ binding sites in the dual promoter, the transcription strength of the dual promoter may be stronger than that of the single promoters. It has been reported that the transcriptional strength of a promoter is not consistent with the number of promoter copies incorporated into the expression system [33]. The transcriptional strength and characteristics of two or more tandem promoters may also depends on cooperation between these single promoters, which might be influenced by their origin [7]. Moreover, apart from the promoter’s transcriptional strength, the extracellular β-CGTase activity is also related to other factors, including the translational efficiency of the mRNA and the secretion efficiency of the signal peptide.

### General applicability of the dual-promoter expression system

The expression strength of the best dual promoter (P_HpaII_–P_amyQ′_), using β-CGTase as the reporter protein, was greater than those of the best single promoter (P_amyQ′_) and the initial promoter (P_amyQ_). To test the general applicability of the dual-promoter system, pullulanase from *Bacillus deramificans* [34] and α-CGTase from *Paenibacillus macerans* JFB05-01 [35] were chosen as reporter proteins. Both of these enzymes are extracellular enzymes used in industrial applications [15, 16]. To accomplish this, the β-CGTase genes in plasmids pHYCGT1, pHYCGT7 and pHYCGTd4, were replaced by the pullulanase gene, yielding plasmids pHYPUL1, pHYPUL7, pHYPULd4, respectively, and by the α-CGTase gene, yielding plasmids and pHYαCGT1, pHYαCGT7, pHYαCGTd4, respectively (Table 2). The six plasmids were used to transform *B. subtilis* CCTCC M 2016536, yielding the six corresponding plasmid-containing strains PUL1, PUL7, PULd4, αCGT1, αCGT7, αCGTd4, respectively (Additional file 1: Table S1).

**Fig. 5:**
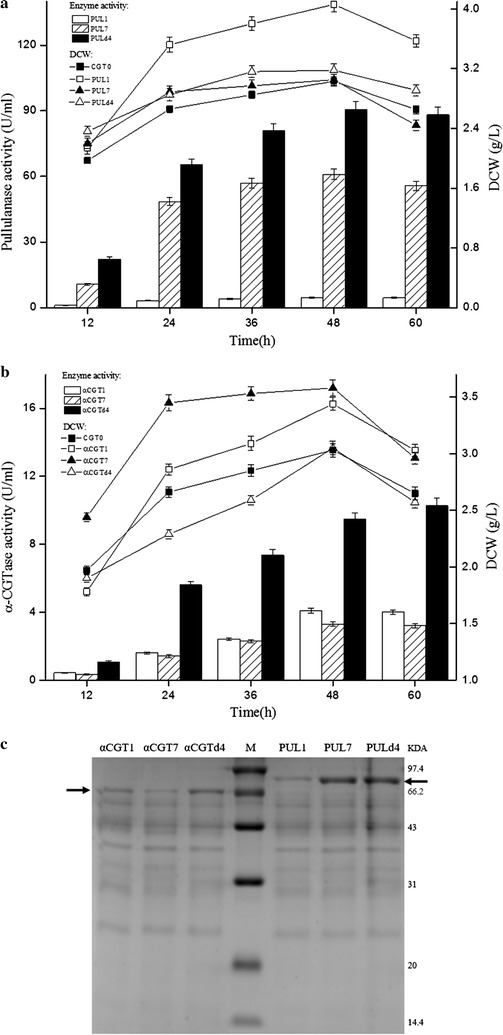
Extracellular expression of pullulanase and α-CGTase mediated by promoters P_amyQ_, P_amyQ′_ and P_HpaII_–P_amyQ′_. Extracellular enzyme activity profile (*column*) and growth curve (*line chart*) of pullulanase expression strains PUL1, PUL7 and PULd4 (**a**), and α-CGTase expression strains αCGT1, αCGT7 and αCGTd4 (**b**). SDS-PAGE analysis of protein expression at 48 h by these plasmid-containing strains (**c**) (*P* < 0.05)

Heterologous protein expression by the plasmid-containing strains was measured using shake-flask cultivations. As shown in Fig. 5a, the pullulanase activities and dry cell weights of plasmid-containing strains PUL1, PUL7 and PULd4 peaked at 48 h. The changes in pullulanase activity were similar among the three plasmid-containing strains. The dry cell weight of plasmid-containing strain PUL1 was higher and changed substantially, compared with that of plasmid-containing strains CGT0, PUL7 and PULd4. Plasmid-containing strain CGT0, which carried an empty expression vector pHY300PLK, showed no pullulanase activity. The extracellular pullulanase activities of plasmid-containing strains PUL1, PUL7 and PULd4 were 4.6, 60.9 and 90.7 U/mL at 48 h, respectively. At this time, plasmid-containing strain PULd4, which harbors the dual-promoter plasmid containing P_HpaII_–P_amyQ′_, showed the highest pullulanase expression; almost 19.7-fold greater than that of PUL1 and 1.5-fold greater than that of PUL7. Furthermore, pullulanase expression by the plasmid-containing strain PUL7, which harbored the single-promoter plasmid containing P_amyQ′_, was 13.2-fold greater than that of PUL1. As shown in Fig. 5c, these result were consistent with an SDS-PAGE analysis of the culture supernatants.

As show in Fig. 5b, the dry cell weights of plasmid-containing strains CGT0, αCGT1, αCGT7 and αCGTd4 peaked at 48 h, and the dry cell weight of plasmid-containing strain αCGT7 was higher than those of plasmid-containing strains CGT0, αCGT1 and αCGTd4. Plasmid-containing strain CGT0, which harbored the empty expression vector pHY300PLK, showed no α-CGTase activity. The α-CGTase activity of plasmid-containing strains αCGT1 and αCGT7 peaked at 48 h, while the α-CGTase activity of plasmid-containing strain αCGTd4 increased 8.1% from 48 to 60 h. At 48 h, the extracellular α-CGTase activities of plasmid-containing strains αCGT1, αCGT7 and αCGTd4 were 4.1, 3.3 and 9.5 U/mL, respectively. As seen with pullulanase, the plasmid-containing strain αCGTd4, which harbored the dual-promoter plasmid containing P_HpaII_–P_amyQ′_ exhibited the highest α-CGTase expression; almost 2.3-fold greater than that of αCGT1 and 2.9-fold greater than that of αCGT7 at 48 h. In contrast to the results with pullulanase and β-CGTase, the α-CGTase expression level of αCGT7, which harbored the single-promoter plasmid containing P_amyQ′_, was weaker than that of αCGT1, which harbored the single-promoter plasmid containing P_amyQ_. These results demonstrate that the dual-promoter P_HpaII_–P_amyQ′_ system not only mediated the highest β-CGTase expression, but also led to highly efficient pullulanase and α-CGTase expression. Thus, this construct is generally applicable for high-level heterologous protein expression. The inconsistent heterologous protein expression levels of promoter P_amyQ′_ with different genes may be due to their different origin and characteristics [7].

### Scale-up of β-CGTase production in a 3-L fermenter

**Fig. 6:**
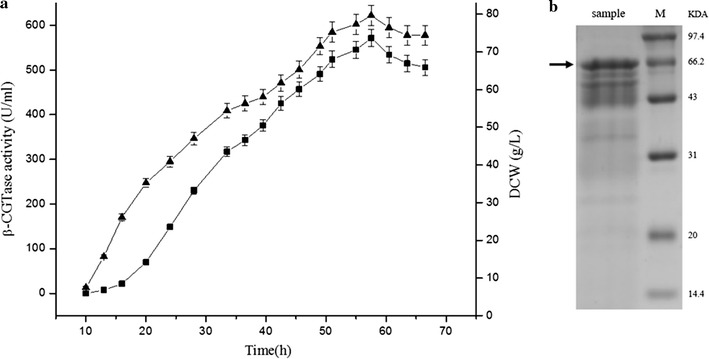
Extracellular β-CGTase expression by plasmid-containing strain CGTd4 in a 3-L fermenter. Extracellular β-CGTase activity (■), Dry cell weight (▲) (**a**). SDS-PAGE analysis of β-CGTase in the 57.5-h culture supernatant (**b**) (*P* < 0.05)

After promoter optimization and general applicability evaluation, the ability of the dual-promoter system to drive β-CGTase production during large-scale fermentation was investigated in 3-L fermenter. In this experiment, we used the plasmid-containing strain CGTd4, which harbored the dual-promoter plasmid containing P_HpaII_–P_amyQ′_. After the inoculation of seed culture, the dissolved oxygen (DO) decreased gradually and the agitation speed was increased correspondingly when DO was below 30%. After about 7 h, there was a sudden increase in DO, along with a decrease in the agitation speed, which signalled that the initial glucose had been consumed. At this time, fed-batch cultivation was started with a feeding speed of 0 to 15 g glucose/h to maintain a glucose concentration of 0.2 to 1.0 g/L. By doing this, we avoided glucose repression. During the fermentation process, the DO was kept at 30%, the pH was kept at 7.0 and the temperature was kept at 37 °C. The extracellular β-CGTase activity of CGTd4 reached 571.2 U/mL after 57.5 h of fermentation (Fig. 6a). This result, which was 18.7-fold greater than the activity obtained during shake-flask cultivation, was verified by SDS-PAGE analysis (Fig. 6b). The dry cell weight reached 79.6 g/L, which was 31.8-fold greater than the cell density seen during shake-flask cultivation.

Although extracellular β-CGTase production has been investigated in prior studies, the yields have been relatively low. The β-CGTase from *Bacillus* sp. G1 was expressed in *Lactococcus lactis* NZ9000, yielding 5.55 U/mL [36]; the β-CGTase from alkalophilic *B. firmus* has been expressed in *E. coli* [37] and alkalophilic *Bacillus* sp [21], yielding 21.6 and 80.12 U/mL, respectively; the β-CGTase from alkalophilic *B. circulans* ATCC21783 was expressed in *B. subtilis*, yielding 210 U/mL [20]. In this study, given that the specific enzyme activity of β-CGTase in *B. subtilis* WB600 was reported to be 231.6 U/mg [17], the β-CGTase expression level of CGTd4 was about 2.5 mg/mL, which was much higher than the yields described in previous studies. This result demonstrates that the dual-promoter P_HpaII_–P_amyQ′_ system has great potential in the industrial production of heterologous proteins. The specific β-CGTase productivity of plasmid-containing stain CGTd4 in 3-L fermenter was 7.18 × 10^3^ U/g DCW, which was lower than the specific β-CGTase productivity seen in shake-flask cultivation (12.2 × 10^3^ U/g DCW). The β-CGTase production level of strain CGTd4 during scale-up may be further improved by optimizing the fermentation medium and feeding strategy. In addition, given the large increase in β-CGTase expression seen in the 3-L fermenter, the expression of pullulanase and α-CGTase may also improve substantially in a 3-L fermenter.

## Conclusion

Using β-CGTase as a reporter protein, nine single-promoter plasmids were constructed and evaluated using shake-flask cultivation. The promoter P_amyQ′_ mediated the highest extracellular β-CGTase activity (24.1 U/mL) under these conditions. Six dual-promoter plasmids were constructed and screened under identical conditions. The dual promoter P_HpaII_–P_amyQ′_ showed the highest extracellular β-CGTase activity (30.5 U/mL) and was relatively glucose repressed. This dual-promoter system was also an excellent mediator of extracellular pullulanase (90.7 U/mL) and α-CGTase (9.5 U/mL) expression, which demonstrates its general applicability. Scale-up of β-CGTase production using the dual-promoter (P_HpaII_–P_amyQ′_) system in a 3-L fermenter resulted in a β-CGTase activity of 571.2 U/mL, which corresponds to an enzyme concentration of 2.5 mg/mL, after 57.5 h cultivation.

## Methods

### Bacterial strains and media

The bacterial strains used in this study are described in Additional file 1: Table S1 *Escherichia coli* JM109 was used as host for genes cloning and plasmids construction. *Bacillus subtilis* CCTCC M 2016536 was used as the host for reporter protein expression. LB medium consisted of 10 g/L tryptone, 5 g/L yeast extract, and 10 g/L NaCl. TB medium contained 12 g/L tryptone, 24 g/L yeast extract, 5 g/L glycerol, 12.54 g/L K_2_HPO_4_, and 2.31 g/L KH_2_PO_4_. The medium used to scale-up β-CGTase production in the 3-L fermenter was modified mineral salt medium [38], which consisted of 20 g/L yeast extract, 2 g/L Na_2_SO_3_, 30 g/L corn steep powder, 10 g/L glucose, 1 g/L MgSO_4_·7H_2_O, 1 g/L (NH_4_)_2_–H-citrate, 2.68 g/L (NH_4_)_2_SO_4_, 4 g/L NaH_2_PO_4_·H_2_O, 14.6 g/L K_2_HPO_4_, and 3 ml/L trace element solution [39]. The feeding solution consisted of 40 mL/L trace element solution, 500 g/L glucose, 63.36 g/L (NH_4_)_2_HPO_4_, and 7.89 g/L MgSO_4_·7H_2_O.

### Plasmids construction and transformation

The characteristics of the plasmids used in this work are presented in Table 2 and the sequences of the primers used during plasmid construction are presented in Additional file 1: Table S2. The plasmid pHYCGT1, which contains the β-CGTase gene from *B. circulans* 251 [40], was constructed on the basis of *B. subtilis*–*E. coli* shuttle vector pHY300PLK (Takara, Dalian, China) as described previously and stored in our laboratory [41]. Plasmids pNCMO2/*pul*A-d2 [24] and pET-20b(+)/*cgt* [42] were previously constructed and are also stored in our laboratory. The single- and dual-promoter plasmids were constructed by using the In-Fusion HD Cloning Plus kit [43], which can ligate two fragments having 15-bp homologous sequences. The single-promoter plasmids was based on plasmid pHYCGT1. Plasmid fragment one, which contained all the sequence of pHYCGT1 except the promoter, was amplified from plasmid pHYCGT1 using primer pair P01/P02. Plasmid fragment two, which contained all the sequence of plasmid pHYCGT1 except the promoter and signal peptide, was amplified from plasmid pHYCGT1 using primer pair P03/P02. Using the corresponding genomic DNA as the template, the promoter fragments P_srf_, P_xyl′_ and P_gsiB_ (*B. subtilis* CCTCC M 2016536), P_xyl_ (*B. megaterium* DSM319; GenBank: CP001982.1) and P_HpaII_ (*Staphylococcus aureus* strain NCCP14562; GenBank: CP013955.1) were amplified using primer pairs P04/P05, P06/P07, P08/P09, P10/P11 and P12/P13, respectively. The promoter and signal peptide fragments P_amyQ′_, P_aprE_, and P_nprE_ were amplified from *B. subtilis* CCTCC M 2016536 genomic DNA using primer pairs P14/P15, P16/P17 and P18/P19. Using the In-Fusion HD Cloning Plus kit, plasmid fragment one was joined with promoter fragments P_srf_, P_xyl′_, P_gsiB_, P_xyl_, and P_HpaII_ to yield plasmids pHYCGT2, pHYCGT3, pHYCGT4, pHYCGT5, and pHYCGT6, respectively. Using the same method, the plasmid fragment two was ligated with the promoter and signal peptide fragments P_amyQ′_, P_aprE_, and P_nprE_ to yield plasmids pHYCGT7, pHYCGT8 and pHYCGT9, respectively.

The dual-promoter plasmids were constructed using the single-promoter plasmid pHYCGT7. Plasmid fragment three was amplified from plasmid pHYCGT7 using primer pair P20/P02. The dual-promoter fragments P_srf_, P_xyl′_, P_gsiB_, P_HpaII_, P_amyQ′_, and P_nprE_ were amplified using plasmids pHYCGT2, pHYCGT3, pHYCGT4, pHYCGT6, pHYCGT7, and pHYCGT9 as their respective templates and primer pairs P04/P21, P06/P22, P08/P23, P12/P24, P14/P25 and P18/P26, respectively. Using the same method used to create the single-promoter plasmids, plasmid fragment three was ligated with the dual-promoter fragments listed above to yield plasmids pHYCGTd1, pHYCGTd2, pHYCGTd3, pHYCGTd4, pHYCGTd5 and pHYCGTd6, respectively.

The pullulanase and α-CGTase expression plasmids were constructed using plasmids pHYCGT1, pHYCGT7 and pHYCGTd4. Plasmid fragments four, five, and six were amplified using plasmids pHYCGT1, pHYCGT7, and pHYCGTd4 as the corresponding templates and primer pairs P27/P28, P27/P29, and P27/P29, respectively. Pullulanase gene fragments one and two were amplified from plasmid pNCMO2/*pul*A-d2 with primer pairs P30/P31 and P32/31, respectively. α-CGTase gene fragments one and two were amplified from plasmid pET-20b(+)/*cgt* using primer pairs P33/P34 and P35/34, respectively. Using the method described above for the single-promoter expression plasmids, gene fragment one was ligated with plasmid fragment four and gene fragment two was ligated with plasmid fragments five and six, respectively. Plasmids pHYPUL1, pHYPUL7, pHYPULd4, which contain the pullulanase gene under control of the P_amyQ_, P_amyQ′_, and dual promotor P_HpaII_–P_amyQ′_, respectively, and plasmids pHYαCGT1, pHYαCGT7, and pHYαCGTd4, which contain the αCGTase gene under control of the P_amyQ_, P_amyQ′_, and dual promotor P_HpaII_–P_amyQ′_, respectively, were constructed as described above. For protein expression, the plasmids constructed above were used to transform *B*. *subtilis* CCTCC M 2016536 competent cells using the method of Anagnostopoulos and Spizizen [44].

### Growth conditions

#### Shake flask cultivation conditions

For routine plasmid construction, *E. coli* JM109 was incubated in LB medium supplemented with 100 mg/L ampicillin for 10 h at 37 °C with shaking at 200 rpm. To express β-CGTase in shake-flask fermentations, *B. subtilis* CCTCC M 2016536 plasmid strains transformed with the appropriate plasmids were incubated in 10 mL of LB medium supplemented with 20 mg/L tetracycline for 10 h at 37 °C with shaking at 200 rpm. A portion (2.5 mL; 5% [v/v]) of this overnight culture was used to inoculate 50 mL of TB medium containing 20 mg/L tetracycline, which was then incubated for 48 h at 30 °C with shaking at 200 rpm. The culture was harvested by centrifugation at 12,000×*g* for 10 min at 4 °C to obtain the culture supernatant, which contained reporter proteins.

#### Fermentation conditions in the 3-L fermenter

Each seed culture was initiated by inoculating 100 mL of LB medium supplemented with 20 mg/L tetracycline in a 500 mL shake flask with 100 μL of glycerol stock (kept frozen at −80 °C). The resulting culture was incubated for 12 h at 37 °C with shaking at 200 rpm. A 3-L fermenter (BioFlo 110, New Brunswick Scientific Co., Edison, NJ) containing 0.9 L fermentation medium that had been adjusted to the optimal state was inoculated with the seed culture (100 mL). After inoculation, the DO decreased gradually and the agitation speed was increased correspondingly when DO was below 30%. After about 7 h, the initial glucose had been consumed and fed-batch cultivation was started with a feeding speed of 0 to 15 g glucose/h to maintain a glucose concentration of 0.2 to 1.0 g/L. During the fermentation process, the DO was kept at 30% by automatically adjusting the air flow rate (1.5 to 4.0 L/min) and agitation speed (300 to 900 rpm). The pH was kept at 7.0 using NH_4_OH and 20% (v/v) H_3_PO_4_ and the temperature was kept at 37 °C. Tetracycline (20 mg/L) was added every 24 h and antifoam was added manually as needed. The culture was sampled at defined time intervals. The samples were centrifuged at 12,000×*g* for 10 min at 4 °C to obtain the culture supernatant.

### Enzyme assays

#### β-CGTase activity

The β-CGTase cyclization activity was measured by adding 0.1 mL of appropriately diluted crude enzyme with 2 mL of preheated 1% (w/v) soluble starch dissolved in 25 mM phosphate buffer (pH 5.5), and then incubating the mixtures at 50 °C for 10 min. The reaction was stopped by adding 0.2 mL of 0.6 M HCl and incubating for an additional 5 to 10 min. At this point, 0.5 mL of 0.6 M Na_2_CO_3_ and 0.2 mL of 1.2 mM phenolphthalein were added sequentially and the mixture was kept at room temperature for 15 min to allow the color to develop. Finally, the absorbance of the assay solution at 550 nm was measured using a spectrophotometer (BioPhotometer plus, Eppendorf Co., Hamburg, Germany). One unit of β-CGTase was defined as the amount of enzyme that formed 1 μmol of β-cyclodextrin per minute from soluble starch under the conditions described above [45].

#### α-CGTase activity

The α-CGTase cyclization activity was measured using an assay similar to that described above for β-CGTase activity. A 0.1 mL aliquot of appropriately diluted crude enzyme was added to 2 mL of preheated 2% (w/v) soluble starch dissolved in 25 mM phosphate buffer (pH 5.5). The resulting mixture was incubating at 50 °C for 10 min. The reaction was stopped by adding 0.2 mL of 3 M HCl and incubating for an additional 5 to 10 min. At this point, 0.2 mL of 0.44 mM methyl orange was added and the mixture was kept at 16 °C for 15 min to allow the color to develop. Finally, the absorbance of the assay solution at 505 nm was measured using a spectrophotometer. One unit of α-CGTase was defined as the amount of enzyme that formed 1 μmol of α-cyclodextrin per minute from soluble starch under the conditions described above [46].

#### Pullulanase activity

Pullulanase activity was measured using a reaction mixture prepared by adding 0.1 mL of appropriately diluted crude enzyme to a preheated mixture of 1 mL 1% (w/v) pullulan dissolved in deionized water and 0.9 mL of 50 mM sodium acetate buffer (pH 4.5). The resulting mixture was incubated at 60 °C for 10 min. The reaction was stopped by adding 3 mL of 3,5-dinitrosalicylic acid solution, boiling for 7 min, and then immediately immersing the reaction in an ice water bath. Finally, 10 mL of deionized water was added and the absorbance of the reaction mixture was measured at 540 nm. One unit of pullulanase activity was defined as the amount of enzyme that released 1 μmol of reducing sugars per minute from pullulan under the conditions described above [47].

### Determination of bacterial biomass

To determine the dry cell weight, 5 mL culture samples were centrifuged at 12,000×*g* for 10 min at 4 °C. These precipitates were resuspended in 0.9% (w/v) NaCl solution and then recentrifuged at 12,000×*g* for 10 min at 4 °C. The resulting precipitates were dried at 105 °C to constant weight.

### SDS-PAGE analysis of reporter proteins

The culture supernatants containing reporter proteins was analyzed using sodium dodecyl sulfate-polyacrylamide gel electrophoresis (SDS-PAGE) using a 12.5% separating gel [48]. 20 ul supernatant was mixed with 5 μL sodium dodecyl sulfate-polyacrylamide gel electrophoresis buffer (5×), after boiling at water bath for 5 mine, 8 μL of the mixture were added to the sodium dodecyl sulfate-polyacrylamide gel. Protein bands were stained with 0.25% Coomassie Brilliant Blue R-250.

### Quantitative real-time PCR

Total RNA was extracted from *B. subtilis* cells using the Simply P Total RNA Extraction Kit (BioFlux, Hangzhou, China). To avoid RNA digestion, all tips and tubes used were RNase-Free. The extracted RNA was treated with the PrimeScript RT reagent Kit with gDNA Eraser (Takara, Dalian, China) to digest genomic DNA and reverse transcribe the cDNA, in that order. Then, the cDNA was used for quantitative real-time PCR (qPCR) to measure the level of β-CGTase gene transcription in different plasmid-containing strains with 16S rRNA as the reference gene. Primer pairs P36/P37 and P38/P39 were used for β-CGTase gene and 16S rRNA gene qPCR amplification, respectively. Using the SYBR Premix Ex Taq II Kit (Takara, Dalian, China), 20 μL qPCR mixture contained 10 μL SYBR Premix Ex Taq II, 0.8 μL forward primer, 0.8 μL reverse primer, 0.4 lb Rod Reference Dye, 2 μL cDNA and 6 μL DEPC-treated water. The qPCR samples were run on an ABI StepOne Real-Time PCR system (Applied Biosystems, San Mateo, CA, USA). The thermal cycling conditions included an initial denaturation step (30 s at 95 °C); 40 cycles of denaturation (5 s at 95 °C) and primer annealing and elongation (30 s at 60 °C); and a melt-curve step (0.3 °C/s, from 60 to 95 °C). The data obtained was analyzed using 2^−ΔΔCT^ methodology [49].

### Statistical analysis

All experiments were conducted independently in triplicate. Data are presented as the average ± standard deviation. Statistical analyses were conducted using Student’s *t* test and differences resulting in *P* < 0.05 were considered statistically significant.

## Additional file

**Additional file 1.** Additional Figures and Tables.

## Abbreviations

β-CGTase: β-cyclodextrin glycosyltransferase
α-CGTase: α-cyclodextrin glycosyltransferase
AprE: alkaline protease
NprE: neutral protease
DO: dissolved oxygen
SDS-PAGE: sodium dodecyl sulfate-polyacrylamide gel electrophoresis
