# Supplementary material for: High-level extracellular protein production in Bacillus subtilis using an optimized dual-promoter expression system
Source: Microb Cell Fact. 2017 Feb 20;16:32. doi: 10.1186/s12934-017-0649-1 (PMC5319110; doi:10.1186/s12934-017-0649-1)
Supplement: Supplementary file 1 — Additional file 1. Additional Figures and Tables. [file 12934_2017_649_MOESM1_ESM.docx]

**Supporting information for**

**High-level extracellular protein production in *Bacillus subtilis* using an optimized dual-promoter expression system**

Kang Zhang^1,2,3^, Lingqia Su^1,2,4^, Xuguo Duan^1,2,5^, Lina Liu^1,2,6^, Jing Wu^1,2,*^

^1^State Key Laboratory of Food Science and Technology, Jiangnan University, 1800 Lihu Avenue, Wuxi, 214122, China.

^2^School of Biotechnology and Key Laboratory of Industrial Biotechnology Ministry of Education, Jiangnan University, 1800 Lihu Avenue, Wuxi, 214122, China.

^3^E-mail: [kangerlang@163.com](mailto:kangerlang@163.com)

^4^E-mail: [sulingqia@126.com](mailto:sulingqia@126.com)

^5^E-mail: [xgduan@jiangnan.edu.cn](mailto:xgduan@jiangnan.edu.cn)

^6^E-mail: [nabestwishes@163.com](mailto:nabestwishes@163.com)

^*^ Corresponding author.

Tel: +86-510-85327802;

Fax: +86-510-85326653.

E-mail: [jingwu@jiangnan.edu.cn](mailto:jingwu@jiangnan.edu.cn)

**Additional figures**

**Figure S1. Cell growth profiles of plasmid-containing strain CGT7 with different concentrations of added glucose.**

**Additional tables**

**Table S1. Parent and plasmid-containing bacterial strains**

| Strain | Properties | Reference |
| --- | --- | --- |
| Parent strains |  |  |
| *E. coli* JM109 | *recA1, thi, endA1, supE44, gyrA96, hsdR17* Δ (*lac-proAB*)/F’[*traD36,proAB^+^, lacІ^q^, lacZ*ΔM15] | Takara |
| *B. subtilis* CCTCC M 2016536 | a gene deficient strain (Δ*srfC*, Δ*spoIIAC*, Δ*nprE*, Δ*aprE*, Δ*amyE*) derived from an undomesticated *B.* *subtilis* strain | CCTCC |
| Plasmid-containing strains |  |  |
| CGT0 | *B. subtilis* CCTCC M 2016536 harboring plasmid pHY300PLK | This work |
| CGT1 | *B. subtilis* CCTCC M 2016536 harboring plasmid pHYCGT1 | This work |
| CGT2 | *B. subtilis* CCTCC M 2016536 harboring plasmid pHYCGT2 | This work |
| CGT3 | *B. subtilis* CCTCC M 2016536 harboring plasmid pHYCGT3 | This work |
| CGT4 | *B. subtilis* CCTCC M 2016536 harboring plasmid pHYCGT4 | This work |
| CGT5 | *B. subtilis* CCTCC M 2016536 harboring plasmid pHYCGT5 | This work |
| CGT6 | *B. subtilis* CCTCC M 2016536 harboring plasmid pHYCGT6 | This work |
| CGT7 | *B. subtilis* CCTCC M 2016536 harboring plasmid pHYCGT7 | This work |
| CGT8 | *B. subtilis* CCTCC M 2016536 harboring plasmid pHYCGT8 | This work |
| CGT9 | *B. subtilis* CCTCC M 2016536 harboring plasmid pHYCGT9 | This work |
| CGTd1 | *B. subtilis* CCTCC M 2016536 harboring plasmid pHYCGTd1 | This work |
| CGTd2 | *B. subtilis* CCTCC M 2016536 harboring plasmid pHYCGTd2 | This work |
| CGTd3 | *B. subtilis* CCTCC M 2016536 harboring plasmid pHYCGTd3 | This work |
| CGTd4 | *B. subtilis* CCTCC M 2016536 harboring plasmid pHYCGTd4 | This work |
| CGTd5 | *B. subtilis* CCTCC M 2016536 harboring plasmid pHYCGTd5 | This work |
| CGTd6 | *B. subtilis* CCTCC M 2016536 harboring plasmid pHYCGTd6 | This work |
| PUL1 | *B. subtilis* CCTCC M 2016536 harboring plasmid pHYPUL1 | This work |
| PUL7 | *B. subtilis* CCTCC M 2016536 harboring plasmid pHYPUL7 | This work |
| PULd4 | *B. subtilis* CCTCC M 2016536 harboring plasmid pHYPULd4 | This work |
| αCGT1 | *B. subtilis* CCTCC M 2016536 harboring plasmid pHYαCGT1 | This work |
| αCGT7 | *B. subtilis* CCTCC M 2016536 harboring plasmid pHYαCGT7 | This work |
| αCGTd4 | *B. subtilis* CCTCC M 2016536 harboring plasmid pHYαCGTd4 | This work |

**Table S2. Primers used in this study.**

| primers | Sequences (5’-3’) |
| --- | --- |
| P01 | ATGATTCAAAAACGAAAGCGG |
| P02 | TCTAGAAGCTTGGGCAAAGC |
| P03 | GCCATGGCACCGGATACCA |
| P04 | GCTTTGCCCAAGCTTCTAGAATCGACAAAAATGTCATGAAAG |
| P05 | TCGTTTTTGAATCATATTGTCATACCTCCCCTAATCTT |
| P06 | GCCCAAGCTTCTAGAAGGTCTTCTTCCGCCACTTG |
| P07 | TCGTTTTTGAATCATTTAAACCACTTTGTTTGCGCT |
| P08 | GCCCAAGCTTCTAGACTAAAAACATTACAGGATATCGCAGT |
| P09 | TCGTTTTTGAATCATTTTGAATTCCTCCTTTAATTGGTG |
| P10 | GCTTTGCCCAAGCTTCTAGAATCAACGTGATATAGGTTTGCTA |
| P11 | TCGTTTTTGAATCATTGTACATTCACCTCCTTGATTT |
| P12 | GCCCAAGCTTCTAGAGTTCGGATTCATCTATGGGAGG |
| P13 | TCGTTTTTGAATCATATGTAAATCGCTCCTTTTTAGGTG |
| P14 | GCCCAAGCTTCTAGAGGCGGCGTTCTGTTTCTGC |
| P15 | ATCCGGTGCCATGGCTTCAGCACTCGCAGCCGC |
| P16 | GCCCAAGCTTCTAGAAGCTGGGTAAAGCCTATGAATTC |
| P17 | ATCCGGTGCCATGGCAGCCTGCGCAGACATGTTG |
| P18 | GCCCAAGCTTCTAGACAGCAGTTCTTTTCCGTCCTC |
| P19 | ATCCGGTGCCATGGCAGCCTGAACACCTGGCAGG |
| P20 | GGCGGCGTTCTGTTTCTGC |
| P21 | AAACAGAACGCCGCCATTGTCATACCTCCCCTAATCTT |
| P22 | AAACAGAACGCCGCCTTAAACCACTTTGTTTGCGCT |
| P23 | AAACAGAACGCCGCCTTCCTCCTTTAATTGGTGTTGGTT |
| P24 | AAACAGAACGCCGCCTAAATCGCTCCTTTTTAGGTGGC |
| P25 | AAACAGAACGCCGCCTCTTGACACTCCTTATTTGATTTTTTG |
| P26 | AAACAGAACGCCGCCAATAAATCCCCCTTTTTGAAAATAC |
| P27 | AAGCTTGGTAATAAAAAAACACCTCC |
| P28 | CATGGCTACGGCTGATGTTT |
| P29 | CATGGCTTCAGCACTCGCAG |
| P30 | TCAGCCGTAGCCATGGATGCAGCGAAACCGGCTGT |
| P31 | TTTATTACCAAGCTTTTACTTTTTACCGTGGTCCG |
| P32 | AGTGCTGAAGCCATGGATGCAGCGAAACCGGCTGT |
| P33 | TCAGCCGTAGCCATGTCACCCGATACGAGCGTGGA |
| P34 | TTTATTACCAAGCTTTTAATTTTGCCAGTCCACCG |
| P35 | AGTGCTGAAGCCATGTCACCCGATACGAGCGTGGA |
| P36 | AATCTGATTGCAGCGGCACA |
| P37 | GTCATACAGGCGACCGTTTT |
| P38 | CATTCAGTTGGGCACTCTAA |
| P39 | TTTGTTCTGTCCATTGTAGC |

**Table S3. β-CGTase expression by different plasmid-containing strains at 48 h.**

| Strains | β-CGTase activity (U/ml) | Dry Cell Weight (g/L) |
| --- | --- | --- |
| CGT1 | 8.5±0.43 | 2.68±0.13 |
| CGT2 | 8.7±0.41 | 2.78±0.14 |
| CGT3 | 9.4±0.45 | 3.31±0.17 |
| CGT4 | 10.5±0.52 | 3.19±0.16 |
| CGT5 | 7±0.3 | 2.26±0.11 |
| CGT6 | 9.6±0.46 | 3.43±0.17 |
| CGT7 | 24.1±1.19 | 2.96±0.13 |
| CGT8 | 6.5±0.32 | 2.91±0.15 |
| CGT9 | 9.3±0.43 | 3.16±0.15 |
| CGTd1 | 23.1±1.16 | 2.39±0.12 |
| CGTd2 | 22.4±1.11 | 3.42±0.17 |
| CGTd3 | 25±1.24 | 2.44±0.12 |
| CGTd4 | 30.5±1.31 | 2.51±0.13 |
| CGTd5 | 28.2±1.20 | 2.53±0.13 |
| CGTd6 | 26.3±1.11 | 2.72±0.14 |

**Table S4. Heterologous enzyme expression by different plasmid-containing strains**

| Strain | Time(h) | Heterologous enzyme activity (U/ml) | Dry Cell Weight (g/L) |
| --- | --- | --- | --- |
| CGT1 | 12 | 1.4±0.16 | 1.86±0.11 |
|  | 24 | 3.74±0.25 | 2.58±0.13 |
|  | 36 | 5.15±0.31 | 2.66±0.12 |
|  | 48 | 8.5±0.43 | 2.68±0.13 |
|  | 60 | 6.08±0.34 | 2.19±0.08 |
| CGT7 | 12 | 3.29±0.33 | 2.06±0.09 |
|  | 24 | 10.3±0.61 | 2.71±0.12 |
|  | 36 | 17.53±0.90 | 2.84±0.12 |
|  | 48 | 24.1±1.19 | 2.96±0.13 |
|  | 60 | 23.66±1.15 | 2.62±0.11 |
| CGTd4 | 12 | 3.14±0.13 | 1.69±0.08 |
|  | 24 | 17.1±0.68 | 2.17±0.11 |
|  | 36 | 18.24±0.73 | 2.35±0.12 |
|  | 48 | 30.5±1.31 | 2.51±0.13 |
|  | 60 | 33.64±1.35 | 2.47±0.12 |
| PUL1 | 12 | 1±0.04 | 2.14±0.18 |
|  | 24 | 3.2±0.13 | 3.52±0.19 |
|  | 36 | 4±0.20 | 3.8±0.17 |
|  | 48 | 4.6±0.23 | 4.06±0.20 |
|  | 60 | 4.5±0.20 | 3.57±0.16 |
| PUL7 | 12 | 10.69±0.43 | 2.2±0.09 |
|  | 24 | 48.5±1.94 | 2.89±0.11 |
|  | 36 | 56.86±2.27 | 2.97±0.13 |
|  | 48 | 60.9±2.54 | 3.05±0.12 |
|  | 60 | 55.61±2.22 | 2.44±0.10 |
| PULd4 | 12 | 22.2±0.89 | 2.36±0.11 |
|  | 24 | 65.33±2.61 | 2.85±0.14 |
|  | 36 | 80.82±3.23 | 3.16±0.13 |
|  | 48 | 90.7±4.03 | 3.18±0.14 |
|  | 60 | 88.23±3.53 | 2.91±0.13 |
| αCGT1 | 12 | 0.43±0.02 | 1.78±0.12 |
|  | 24 | 1.61±0.05 | 2.86±0.13 |
|  | 36 | 2.41±0.09 | 3.09±0.16 |
|  | 48 | 4.1±0.16 | 3.44±0.16 |
|  | 60 | 4±0.13 | 3.03±0.14 |
| αCGT7 | 12 | 0.35±0.04 | 2.44±0.14 |
|  | 24 | 1.42±0.09 | 3.45±0.17 |
|  | 36 | 2.3±0.09 | 3.53±0.16 |
|  | 48 | 3.3±0.13 | 3.58±0.17 |
|  | 60 | 3.2±0.11 | 2.96±0.15 |
| αCGTd4 | 12 | 1.08±0.04 | 1.9±0.09 |
|  | 24 | 5.61±0.20 | 2.29±0.10 |
|  | 36 | 7.36±0.34 | 2.59±0.10 |
|  | 48 | 9.5±0.38 | 3.05±0.14 |
|  | 60 | 10.27±0.46 | 2.57±0.12 |

**Table S5. Effect of glucose addition on β-CGTase expression by plasmid-containing strains CGT7 and CGTd4.**

| Strain | Glucose concentration (%) | β-CGTase activity (U/ml) | Dry Cell Weight (g/L) |
| --- | --- | --- | --- |
| CGT7 | 0 | 24.1±1 | 2.96±0.14 |
|  | 1 | 20±1 | 2.42±0.11 |
|  | 1.5 | 8.7±0.42 | 2.17±0.11 |
|  | 2 | 7.1±0.33 | 2.23±0.10 |
|  | 2.5 | 1.8±0.08 | 2.48±0.11 |
| CGTd4 | 0 | 30.5±1.32 | 2.51±0.13 |
|  | 1 | 21.53±1.01 | 2.23±0.11 |
|  | 1.5 | 17.83±0.79 | 2.37±0.12 |
|  | 2 | 15.25±0.65 | 2.40±0.12 |
|  | 2.5 | 9.73±0.41 | 2.41±0.12 |
